# Intradialysis hypotension and hypertension in patients with end stage kidney disease in Nigeria: risk factors and clinical correlates

**DOI:** 10.4314/gmj.v55i1.6

**Published:** 2021-03

**Authors:** Peter K Uduagbamen, Solomon Kadiri

**Affiliations:** 1 Division of Nephrology and Hypertension, Department of Internal Medicine, Babcock University Teaching Hospital, Ilishan-Remo, Nigeria; 2 Nephrology Unit, Department of Internal Medicine, Federal Medical Centre, Abeokuta, Nigeria; 3 Nephrology Unit, Department of Internal Medicine, University College Hospital, Ibadan, Nigeria

**Keywords:** End stage kidney disease, intradialysis hypotension, intradialysis hypertension

## Abstract

**Background:**

Many shortcomings associated with haemodialysis for instance, intradialysis blood pressure changes, often lead to inadequate dialysis dose. Measures are needed to improve on this.

**Objectives:**

To determine the risk factors and clinical correlates of intradialysis blood pressure variations.

**Methods:**

Maintenance haemodialysis sessions for 232 consented patients with end stage kidney disease who had 1248 sessions were studied. Data collected was from history, examination findings, serum electrolytes and hematocrit. Blood pressure reading was taken manually at rest. Statistical analysis was with SPSS 22. Chi square and t-test were used to compare proportions and means respectively while regression analysis was used to determine predictors of blood pressure changes.

**Results:**

The mean age of participants was 49.9 ± 4.6. More participants (38.8%) had hypertension associated CKD, than chronic glomerulonephritis, (37.9%). Majority (60.7%) had internal jugular catheter. Intradialysis hypertension was commoner than intradialysis hypotension (24.4% versus 19.4%). Intradialysis hypotension was commoner in females, diabetics and with less frequent dialysis while intradialysis hypertension was commoner in males, frequent erythropoietin use. The mean dialysis dose (Kt/V) was 1.02 ± 0.4, with 0.68 ± 0.1 for intradialysis hypotension and 0.84 ± 0.2 for intradialysis hypertension.

**Conclusion:**

Risk factors for intradialysis hypertension were males, frequent erythropoietin use while for intradialysis hypotension, were female gender and less frequent dialysis. Effective intra and inter-dialytic blood pressure control with adequate pre dialysis work up should be carried out to lessen the degree, burden and outcome of these variations.

**Funding:**

None declared

## Introduction

Effective peridialysis blood pressure and other cardiovascular function control is necessary for the delivery of an adequate dialysis dose and attainment of optimal clinical outcome.^1^ The blood pressure (BP) is a key indicator of extracellular volume (ECV) and an index of the cardiac reserve in health and disease.^2^ A very high prevalence of hypertension (HTN) and other cardiovascular diseases is reported amongst CKD patients, particularly in end stage, with 57% of the hypertensive population in Ghana found to have poorly treated hypertension.^3^ Intradialysis hypotension (IDH) and intradialysis hypertension (IDHT) are reported to be quite common in Nigeria and many low income countries (LICs), interruptions or discontinuation of dialysis from these could lead to suboptimal dialysis doses, reduced quality of life (QOL) and increased morbidity and mortality.^4^ Despite the reported high prevalence of IDH and IDHT in LICs, there is paucity of literature on the risk factors associated with them. We therefore determined the risk factors and clinical correlates of intradialysis blood pressure variations.

## Methods

This was a hospital based two centre descriptive, cross sectional study in which consecutive sampling method was used in recruiting two hundred and thirty two (143 males and 89 females) participants with CKD, in end stage, according to KDOQI 2012 criteria.^5^ All participants gave informed consent and thereafter underwent 1248 haemodialysis (HD) sessions with each participant undergoing a maximum of six sessions.

The study lasted for 18 months (12 in centre A and 6 in centre B). Eighty eight were excluded on account of transplantation, been less than 18 years, pelvic tumours, infections, dialysis lasting less than three hours, or less than once weekly. Data was taken from history and patients' case notes and variables retrieved were age, gender, history of preceding pharyngitis or skin sepsis, aetiology of CKD, duration of each dialysis session and postdialysis weight for preceding session.

The height was taken without shoes and weight on very light clothing using standardized scales and body mass index (BMI) was calculated. For each session, the interdialytic weight gain (IDWG) was taken as the difference between the predialysis weight for the index session and the post dialysis weight of the preceding session. The IDWG, in addition to clinical parameters such as BP, primary cause of CKD and class of antihypertensives, guided the prescription of each session's dose, particularly the target ultrafiltration volume (UFV) and blood flow rate (BFR). Predialysis temperature, pulse rate (PR), BP and percentage oxygen saturation (SPO_2_] were taken after five minutes of rest. All participants had normal temperatures at the commencement of each session. Intradialysis, for every temperature increase of up to 1°C, concurrent with increase in PR of up to 120 beats/minute, and a reducing blood pressure, the BFR was reduced by 50 ml/min to reduce the risk of IDH and possible arrhythmias. All BP readings were taken manually with participants lying supine. Two predialysis blood samples were taken for analysis of the serum electrolyte, urea and creatinine, and the haematocrit (HCT). At the first session for each participant, pre dialysis blood sample was taken to determine the serum albumin. The vital signs were repeated half hourly throughout dialysis, and when IDH or IDHT occurred, vital signs were taken quarter hourly.

### Definitions

The post dialysis weight was defined as the pre dialysis weight plus administered fluids and blood minus the ultrafiltration volume.

IDH: Systolic blood pressure fall of ≥20 mmHg with symptoms according to the European Best Practices Guidelines (EBPG).^6^,^7^ but without nursing intervention.

IDHT: Systolic blood pressure rise of >10 mmHg.^8^ In this study, for simplicity and convenience, the adequacy of the delivered dialysis dose was classified as: Normal (Kt/V ≥1.2 and URR ≥65.0%), Low (Kt/V 0.9–1.1 and URR 50.0–64.9%) or Very Low (Kt/V <0.9 and URR <50.0%).

Kidney biopsy was not used in classifying the cause of CKD. Hypertension associated CKD was defined as kidney disease arising from long standing hypertension, prevalent from late middle age upwards while chronic glomerulonephritis (CGN) was defined as kidney disease that led to hypertension, common in the young and early middle age, with or without preceding history of pharyngitis or skin sepsis.

Patency of the internal jugular access was checked by withdrawing 1ml of blood, predialysis samples were taken and both arterial and venous ends were flushed with heparinized saline. Blood was taken from femoral catheters immediately fresh ones were sited, and with arteriovenous fistula (AVF), samples were taken from a peripheral vein in the contralateral arm. For participants with increased risk of bleeding, heparin dose was reduced or withheld depending on the degree of the clotting profile derangement. Participants were connected through the arterial and then the venous portal. Where the BFR was altered, for instance, reductions to manage IDH or increases to improve the dialysis dose, the mean was calculated, taking into consideration, the duration of alteration. The dialysate flow rate (DFR) was 500ml/min for all sections.

The stop dialysate flow method was used in post dialysis blood sampling. At the end of dialysis time, dialysate flow was stopped and blood pump flow continued.^9^ Five minutes after stopping the dialysate flow, blood was taken from the arterial portal, first, for the serum biochemistry (minimizes access recirculation) and then HCT. The urea reduction ratio (URR) was calculated, and Kt/V was calculated using the Daugirdas second generation logarithmic estimation of single pool using the predialysis urea, post dialysis urea, UFV, post dialysis weight and dialysis duration.^10^

Niprol Surdial X machines were used in 1134 HD sessions and 5008 Fresenius machines in 114 sessions. The sample for serum albumin was analyzed using the bromocresol green method. It overestimate by (about 3.5g/dl) the serum albumin in renal disease including dialysis treatment and other cases of hypoalbuminaemia.^11^ Cut-off values for normal serum albumin, when it is used, may necessarily need be raised by about 3–3.5 or 5.5–7 compared to the bromocresol purple or the immunophelometric assay respectively.

Data generated from the study was analyzed using SPSS 22. Continuous variables were presented as means with standard deviation and compared using t-test while categorical variables were presented as proportions and compared using Chi square test or Fisher's exact test. The P-value <0.05 was considered statistically significant. ANOVA was used to compare three or more variables. Multivariate regression analysis was carried out to determine the predictors of IDH and IDHT.

This study was approved by the Human Ethics Committees of the Federal Medical Centre, Abeokuta and Babcock University, Ilishan-Remo (FMCA/470/HREC/03/2017, NHREC/08/10-2015) and (BUHREC/723/19, NHREC/24/01/2018).

## Results

Two hundred and thirty two participants (143 males, 89 females) who had 1248 HD sessions were studied. The mean age of participants was 49.9 ± 4.6 yrs. Fifty three (22.8%) were between 18 and 39 years, 111 (47.8%) were between 40 and 59 years and 68 (29.4%) were at least 60 years (Table 1). A greater proportion of participants, 90 (38.9%) had hypertension associated CKD, and all participants were taking hypotensive drugs. Dialysis treatment was sponsored by family 100 (43.1%), self, 85 (36.6) and by institutions 47 (20.3%). A greater proportion 92 (39.7%) of participants were taking three different hypotensive drugs, 87 (37.5%) were taking two and 53 (22.8%) were using only one.

**Table 1:** Socio-demographic and clinical characteristics of participants

Variables	Frequency N=232 (%)	Dialysis sessions N=1248 (%)
**Gender** **Males** **Females**	143 (61.6) 89 (38.4)	818 (65.5) 430 (34.5)
**Age, years** **18–39** **40–59** **≥60**	53 (22.8) 111 (47.9) 68 (29.3)	324 (26.0) 697 (55.8) 227 (18.2)
**Aetiology of CKD** **Hypertension** **CGN** **Diabetics** **Others**	90 (38.9) 88 (37.9) 27 (11.6) 27 (11.6)	544 (43.6) 446 (35.7) 125 (10.0) 133 (10.7)
**BMI, kg/m^2^** **<19.5** **19.5–24.9** **≥25.0**	7 (3.0) 103 (44.4) 122 (52.6)	35 (2.8) 571 (45.8) 642 (51.4)

CKD - chronic kidney disease,

CGN-chronic glomerulonephritis, BMI-body mass index

The mean BMI was 23.5 ± 4.2 kg/m^2^. There was a progressive and significant reduction in mean blood pressure throughout dialysis, (P<0.001). There was also a significant rise in the SPO_2_ throughout dialysis, (P=0.02). The BP of many participants reduced and normalized as dialysis progressed, P=0.03. Episodes of IDHT, (24.4%) were more than IDH (19.4%). Males had most (67.9%) of IDHT while females and the elderly had most (59.9%) of IDH.

Most of the IDH occurred before the second intra-dialytic hour (mean 64 ± 3.8 minutes) while most of the IDHT occurred after the second intra-dialytic hour (mean 146 ±7.1 minutes). Of the sessions with IDH and IDHT, dialysis was terminated in 3.3% and 0.3% respectively, with one intra dialysis cardiac arrest and death associated with IDHT. There were significant differences between the mean predialysis and postdialysis sodium (P=0.01), potassium (P=0.001), chloride (P=0.002), bicarbonate (P=0.001), calcium (P=0.001), phosphate (P=0.001), urea (P<0.001), creatinine (P<0.001) and eGFR 0.02, (Table 2). A greater proportion of participants had weekly dialysis. Majority (78.4%) of the dialysis machines were Nipro SURDIAL TM X, 92.3% of all dialysis sessions lasted for four hours and 97.4% of all sessions were with high flux dialyzers. Bicarbonate based dialysate was used in all sessions.

**Table 2:** Laboratory results of participants

Variables	Pre dialysis Mean (SD)	Post dialysis Mean (SD)	t-test	P-value
**Sodium, mmol/l**	128.8 (6.7)	134.4 (5.8)	1.4	0.01
**Potassium, mmol/l**	5.6 (1.2)	4.2 (0.8)	5.8	0.001
**Bicarbonate,** **mmol/l**	17.4 (3.6)	20.6 (6.2)	5.7	0.001
**Chloride, mmol/l**	99.5 (7.8)	101.6 (9.2)	4.4	0.002
**Calcium, mmol/l**	2.0 (1.1)	2.2 (1.2)	4.9	0.001
**Phosphate, mmol/l**	1.94 (1.4)	1.6 (0.7)	2.2	0.001
**Urea, mmol/l**	16.4 (2.3)	8.7 (3.6)	10.2	<0.001
**Creatinine, umol/l**	588.6 (23.6)	302.9 (11.7)	9.5	<0.001
**Haematocrit, %**	25.5 (5.2)	25.9 (7.3)	0.9	0.08

The mean UFV was 1.3L, 1.5L for females and 1.2L for males. Majority (50.5%) of the dialysis sessions had UFV <2L Two hundred and seventy seven (22.2%) sessions were done at BFR ≥350 ml/min, 664 (53.2%) at 250–349 ml/min and 307 (24.6%) sessions at BFR 150–249 ml/min. The mean Kt/V and URR for the study were 1.02 ± 0.4 and 51.7 ± 4.2% respectively. Dialysis dose was adequate in 115 (9.2%) sessions, low in 505 (40.5%) and very low in 628 (50.3%) sessions.

The prevalence of IDHT was higher than IDH (24.4% vs. 19.4%). Males had more sessions with IDHTthan females, P=0-002. IDH was common in the young and elderly while IDHT was common in the middle aged, P=0.001. The number(s) of blood pressure reducing drugs increased with risk of IDHT, P<0.001. The frequency of erythropoietin use was directly related to the risk for IDHT, P<0.001. Most episodes of IDH occurred within the first two intradialytic hours (mean 64 ± 3.8 minutes) while most episodes of IDHT occurred after the second intradialytic hour (mean 146 +7.1 minutes). Dialysis was terminated in 3.3% of the IDH episodes and 0.3% of the IDHT and there was a case of intra dialysis cardiac arrest and death associated with IDHT.

The BMI was inversely related to the risk of developing IDH and directly related to the risk of developing IDHT, P-0.001. The mean systolic and diastolic BP, predialysis, two hours intradialysis and postdialysis were 168.2 ± 5.6 mmHg and 96.4 ± 3.2) mmHg, 142.3 ± 5.2 mmHg and 88.7 ± 6.3 mmHg, and 138.2 ± 8.2 mmHg and 73.5 ± 4.7 mmHg, and P=0.001 and P=0.001 respectively. The mean SPO_2_, predialysis, two hours intradialysis and postdialysis were 93.1 ± 8.4%, 94.8 ± 6.9% and 97.7 ± 6.6% respectively, P=0.002. Lower predialysis serum creatinine was more associated with IDHT than IDH, P<0.001.

Predialysis serum albumin was directly related to the risk of developing IDHT as against its inverse relationship with IDH, P=0.002. The BFR was positively correlated with risk for IDH and negatively correlated with risk for IDHT, P=0.01. Participants carrying the internal jugular catheter and AVF had more IDH than IDHT, P=0.001. Dialyzer size was positively related to the risk for IDH, P=0.01. The UFV was directly related to the risk for IDH, P<0.001. From multivariate regression analysis (Table 4), completed dialysis sessions, BFR <350ml/min, dialyzer SA ≤1.4m^2^, and UFV <1Litre predicted intradialysis BP changes.

**Table 4:** Multivariate regression analysis showing predictors of intradialysis blood pressure changes

Variable	OR	95% CI	P-value
**Advancing age**	1.68	1.05–2.66	0.06
**Elevated Creatinine**	0.03	0.0025–0.028	0.03
**Glomerular filtration rate**	9.46	1.13–70.22	0.002
**Haematocrit <33%**	4.52	3.03–7.01	0.001
**Blood flow rate <350 ml/min**	0.03	0.031–0.034	0.04
**Dialysis duration <4 hours**	3.32	3.18–3.46	0.01
**Ultrafiltration rate < 1 Litre**	5.24	2.46–9.52	0.001
**Femoral access**	0.02	9.91–0.04	0.03
**Dialyzer surface area <1.7m^2^**	4.26	3.76–4.88	0.04
**Diabetes**	1.74	1.08–262	0.05
**Serum albumin <35g/dl**	3.94	3.33–5.07	0.001

OR- odd ratio, CI-confidence interval

## Discussion

The prevalence of IDH (19.4%) was lower than that of IDHT (24.4%). The lower prevalence of IDH can partly be attributed to the inclusion of symptoms in its diagnostic criteria unlike IDHT. This prevalence is however higher than that reported by Kiupers et al who found a prevalence of 8.5% using the EBPG that included nursing interventions (unlike this study) in its diagnostic criteria.^6^, ^7^ However the prevalence in our study mirrors that by Okaka *et al* in Nigeria who reported a prevalence of 19.8% in a retrospective study in which symptom manifestation was not included in the diagnostic criteria.^4^ The non-uniformity of diagnostic criteria accounted for the wide range of IDH prevalence from various studies.^12^,^13^.

The reliability or otherwise of symptoms reportage by patients during dialysis could also compound the challenges associated with determining the true prevalence of IDH as the subjective assessment of these symptoms varies widely among dialysis patients.^14^

The prevalence of IDHT in this study is similar to the 22.3% reported by Van Burren et al.^15^ In the CLIMB study, Inrig et al found a 2.17 fold increase risk for a combined end-point mortality and occurrence of cardiovascular event, (HR-2.17, (95% CI-1.13–4.15) among patients with intradialysis hypertension.^16^ The higher prevalence of IDH and IDHT in LICs is suggestive of a higher all-cause mortality and cardiovascular events in their dialysis population. Diuretics reduce the extraction ratio, however, the combination of diuretic overuse, excessive ultrafiltration, poor cardiac reserve and autonomic neuropathy is responsible for most incidences of IDH.^17^ Intra dialysis BP changes correlates with ongoing cardiac systolic activity, heart rate (HR), cardiac output (CO) and stroke volume (SV), and could alter the prescribed BFR, response to ultrafiltration, and affect the immediate and short-term dialysis outcome.^18^ In conditions associated with compromised cardiac output, volume replacement during the initial steep slope of the Frank Sterling Curve (FSC) augments the cardiac systolic and diastolic function resulting in increased stroke volume, cardiac output and blood pressure, preventing a precipitous fall in BP.

However, fluid replacement following intra dialytic ultrafiltration, in the plateau phase of the FSC leads to interstitial fluid retention thereby reducing the intravascular volume that can induce myocardial hypoperfusion, which when recurrent, leads to myocardial stunning with attendant poor QOL, high morbidity and mortality.^19^

More males than females participated in the study, similar to findings from earlier studies in Nigeria where males seek health care more than females, associated with cultural practices and gender bias.^4^, ^20^ Hypertension, a major cause of CKD, being commoner in males is also contributory.^21^ The higher risk of IDH in females and IDHT in males mirrors findings by Stefansson et al. 22 The relative poor responsiveness of males to inhibition of the RAAS favors a faster decline of kidney function in males with CKD^23^ The large middle aged population in our study is not in agreement with a previous study in Nigeria that reported that CGN (commonly found in the young), was the commonest cause of CKD.^24^ Our finding is also not in agreement with those from the western nations where Chou et al reported that CKD is commoner in the elderly population than in the young or middle aged.^25^ The greater awareness of the need to treat infections promptly and fully in our clime could be responsible for the shift as infective organisms causing acute glomerulonephritis and/or pyelonephritis appear to be better eradicated now. Our findings however agrees with Okaka et al who found more cases of hypertension than CGN as cause of CKD further buttressing this shift.^4^

The inverse relationship between age and the risk for IDH agrees with findings by Morimotor et al.^26^, Primary glomerular disease are known to have tubulointerstitial component and tend to manifest with more fluid retention. The diminished glomerular filtration (the initiating stage of urine formation) cause increased inter dialytic weight gain (from excessive fluid retention) and this is commonly managed with large volume ultrafiltration which can precipitate IDH.^27^

The use of erythropoiesis stimulating agents (ESAs) increased the risk for IDHT, similar to findings by Wilson et al that linked the use of ESAs with hypertension, hyperviscousity and thrombus formation.^28^ The positive correlation between BMI and IDHT in this study, mirrors findings by Shu-Zhong.^29^ Obesity in itself is a risk factor for the development of hypertension.^30^ Obesity causes increased tubular sodium absorption and suppression of natriuresis leading to increased cardiac output and blood pressure.

Lower predialysis blood pressures were directly related to IDH as higher pressures were risk for IDHT similar to report by Chang el tal.^31^. Extra corporal fluid removal intradialysis could lead to a precipitous fall in blood pressure but with higher predialysis blood pressures, the presence of precipitants of blood pressure reductions may just result in BP normalization.^32^

Low serum sodium was associated with high risk of IDH as higher sodium levels were associated with increased risk of IDHT similar to findings by Dahlmann et a.^33^ Sodium efflux from the cell coupled with potassium entry (mediated by the Na^+^K^+^ATPase) causes obligatory passive calcium influx followed by depolarization, increased inotropy, chronotropy, and blood pressure. Patients with end stage kidney disease commonly have left ventricular (LV) remodeling with concentric hypertrophy which could be associated with reduced LV filling pressures during dialysis. This causes reduction in preload and stroke volume. The normal compensatory response of increased myocardial contractility and heart rate to low stroke volume is depressed in dialysis patients due to cardiac remodeling, and this could lead to low cardiac output and precipitate IDH.^34^ Alteration in the dialysate sodium concentration has become a means by which these inter compartmental fluid changes can be regulated.^35^

Metabolic acidosis increased the risk of IDH in this study, similar to findings by Meyring-Wosten et al.^36^ Acidosis causes cutaneous vasodilatation and reduces the effective blood volume and BP, and if severe, can cause myocardial irritation, adrenergic overactivity, blood pressure increase and arrhythmias.^37^ However, the introduction of bicarbonate as dialysate buffer has reduced the degree of acidosis and its effects.^38^ Though, respiratory alkalosis is uncommon in CKD, it is worth noting that the vasodilatation seen in dialysis patients could not always be attributable to metabolic acidosis but also to respiratory alkalosis. The DOPPS study found an increase all-cause mortality with high dialysate bicarbonate.^39^ Hypobicarbonaemia in dialysis patients from increased protein intake, interdialytic weight gain, and Sevalamer use, is managed by ultrafiltration which increases serum bicarbonate from the contraction of the bicarbonate space.^40^

The negative correlation between predialysis creatinine and intradialysis BP levels reported by Van Buren et al.^41^ The lesser intradialysis osmolar changes lessens the need for high UFR in them and the lesser edema reported in them is in keeping with this narrow intradialysis osmotic gradient. Lower predialysis creatinine suggest more frequent dialysis, significant residual kidney function (GFR ≥5ml/min) and a relatively preserved cardiac function that could respond to vasopressor stimulation that would lead to IDHT. It is worth noting that in this study, participants with severe dialysis cachexia from chronic dialysis and protein energy malnutrition (PEM) were on many occasions dialyzed on account of severe acidosis, excessive fluid retention and electrolyte derangement with little consideration of the serum creatinine level.

In this study, hypoalbuminaemia increased the risk for IDH due to poor intravascular filling. 42 Lower HCT was associated with increased incidence of intradialysis hypertension similar to findings by Raikou et al.^43^ Higher ultrafiltration in this study was associated with higher dialysis dose, similar to findings by Assimon et al who also reported that greater than 10–13ml/hr/kg was associated with increase mortality and cardiovascular events.^44^

The use of AVF was associated with a greater tendency to developing IDH in this study. Saleh et al^45^ reported that the creation of AVF in dialysis patients result in reduced peripheral resistance. The AVF increases the risk for pulmonary arterial hypertension, acute decompensated heart failure and steal syndrome from increase blood volume in the right ventricles coming from the superior vena cava (SVC). It can also slow down the rate of kidney function decline in CKD sufferers. 45

Dialysis sessions lower than four hours were more likely to result to IDH than IDHT and this agrees with findings by Van Burren et al.^44^ A larger proportion of the UFV is removed in the first half of the routine four hours dialysis. As dialysis continues with low UFR, the continued activation of the vasopressor system can lead to increases in blood pressure, high enough to meet the definition of IDHT (SBP increase of ≥10 mmHg). Overshooting of the SNS and RAAS can cause myocardial hyper contractility and irritation which can induce malignant ventricular tachyarrhythmia with intradialysis cardiac arrest.^46^.

The management of IDH involves saline infusion, cooling the dialysate solution, reducing UFV and BFR and if persistent, the use of inotropes. IDHT could precipitate arrhythmias during dialysis as it could lead to myocardial ischemia and even infarction as seen in one of the participants in the study. Management involves increasing the BFR, the UFV and the use of antihypertensive drugs, mild sedatives and antiarrhythmic agents.^43^

Some limitations were encountered in this study such as the non-availability of the automated blood pressure monitor (ABPM), useful in blood pressure dynamics in the interdialytic period. We could not determine the dry weight which would have better guided the formulation of the dialysis prescription. We were also unable to determine the residual kidney function which would have enabled us to determine the relative contribution of the kidneys to the delivered dialysis dose. Though it was a two center study, further studies are still needed to ascertain racial and environmental contribution to the delivered dose and clinical outcome.

However, being a more comprehensive study that considered the patient, the disease process, laboratory results, dialysis personnel and facilities related factors, this will brings to the fore, various factors which when effectively manage will lead to better dialysis outcome for the teaming dialysis population.

## Conclusion

Intradialysis hypotension and hypertension are common findings in our environment with prevalence of 19.4% and 24.4% respectively. They are associated with suboptimal dialysis doses and poor patient outcome. Risk factors for IDH identified in this study include female gender, advanced age, diabetes, acidosis, and for IDHT included middle age, use of ≥3 antihypertensive drugs, lower predialysis creatinine, frequent erythropoietin use and male gender. Good blood pressure control coupled with patients' adherence to treatment regimen are important measures to curtail these events. An effective predialysis workup coupled with an all-encompassing dialysis prescription are needed to minimize these factors and their attendant consequences on patient outcome.

## Figures and Tables

**Table 3:** Relationship between participants characteristic and intradialysis blood pressure changes

Variables	All sessions N=1248 (%)	Insignificant BP change N=701 (%)	IDH N=242 (%)	IDHT N=305 (%)	P-value
**Gender** **Males** **Females**	818 (65.5) 430 (34.5)	422 (60.2) 279 (39.8)	141 (58.3) 101 (41.7)	255 (83.6) 50 (16.4)	0.02
**Age, years** **18–39** **40–59** **>60**	324 (25.9) 697 (55.9) 227 (18.2)	175 (25.0) 410 (58.5) 116 (16.5)	78 (32.4) 92 (38.0) 72 (29.6)	71 (23.3) 195 (63.8) 39 (12.9)	0.001
**CKD Aetiology** **Hypertension** **CGN** **Diabetes** **Others**	544 (43.6) 446 (35.7) 125 (10.0) 133 (10.7)	309 (44.1) 266 (37.9) 61 (8.7) 65 (9.3)	86 (35.5) 72 (29.8) 48 (19.8) 36 (14.9)	149 (48.9) 108 (35.4) 16 (5.2) 32 (10.5)	0.001
**BMI, kg/m^2^** **<19.5** **19.5–24.9** **≥25.0**	42 (3.3) 651 (52.2) 555 (44.5)	21 (3.0) 362 (51.6) 318 (45.4)	16 (6.6) 161 (66.5) 65 (26.9)	5 (1.6) 128 (42.0) 172 (56.4)	<0.001
**Pre Systolic BP, mmHg** **<140** **>140**	263 (21.1) 985 (78.9)	129 (18.4) 572 (81.6)	116 (48.1) 126 (51.9)	18 (6.0) 287 (94.0)	0.01
**PreD Diastolic BP, mmHg** **<90** **≥90**	167 (13.4) 1081 (86.6)	80 (11.4) 621 (88.6)	45 (18.5) 197 (81.5)	42 (13.8) 263 (86.2)	0.01
**PreD SPO_2_, %** **<95** **>95**	1159 (92.9) 89 (7.1)	643 (91.7) 58 (8.3)	240 (99.1) 2 (0.9)	276 (90.5) 29 (9.5)	<0.001*
**PreD Sodium, mmol/l** **<135** **135–145** **>145**	938 (75.1) 263 (21.1) 47 (3.8)	573 (81.7) 110 15.7) 18 (2.6)	194 (80.5) 43 (17.6) 5 (1.9)	171 (56.0) 110 (36.2) 24 (7.8)	0.01
**PreD Potassium, mmol/l** **<3.5** **3.5–5.0** **>5.0**	8 (0.6) 52 (4.2) 1188 (95.2)	4 (0.5) 46 (6.6) 651 (92.9)	3 (1.2) 6 (2.5) 233 (96.3)	1 (0.3) 0 (0.0) 304 (99.7)	0.001*
**PreD Bicarbonate, mmol/l** **<20** **20–30** **≥30**	931 (74.6) 193 (15.5) 124 (9.9)	622 (88.7) 51 (7.3) 28 (4.0)	159 (65.7) 47 (19.5) 36 (14.8)	150 (49.2) 95 (31.0) 60 (19.8)	<0.001
**PreD Creatinine, umol/l** **<110** **110–499** **≥500**	124 (10.0) 398 (31.9) 726 (58.1)	26 (3.7) 139 (19.8) 536 (76.5)	8 (3.3) 83 (34.3) 151 (62.4)	90 (29.5) 176 (57.7) 39 (12.8)	<0.001
**PreD Urea, mmol/l** **<3.0** **3.0–7.0** **>7.0**	6 (0.5) 382 (30.6) 860 (68.9)	3 (0.4) 152 (21.7) 546 (77.9)	1 (0.4) 35 (14.4) 206 (85.2)	2 (0.7) 195 (63.9) 108 (35.4)	<0.001*
**PreD Albumin, mg/dl** **<28** **28–35** **>35**	663 (53.1) 462 (37.0) 123 (9.9)	376 (53.6) 267 (38.1) 58 (8.3)	137 (56.6) 87 (36.1) 18 (7.4)	150 (49.2) 108 (35.4) 47 (15.4)	0.002
**PreD Haematocrit, %** **<33** **33–36** **>36**	1033 (82.8) 179 (14.3) 36 (2.9)	563 (80.3) 114 (16.3) 24 (3.4)	199 (82.2) 33 (13.7) 10 (4.1)	271 (88.9) 32 (10.5) 2 (0.6)	0.004*
**BFR, ml/min** **200–299** **300–399** **≥400**	307 (24.6) 663 (53.1) 278 (22.3)	205 (32.0) 378 (54.6) 118 (13.4)	34 (13.9) 116 (48.1) 92 (38.0)	68 (22.4) 169 (55.2) 68 (22.4)	0.01
**Dialyzer area, m^2^** **1.4** **1.7/1.8**	6 (0.5) 1242 (99.5)	3 (0.4) 698 (99.6)	0 (0.0) 242 (100.0)	3 (0,9) 302 (99.1)	0.001*
**Access** **Femoral** **Int jugular** **Arteriovenous fistula**	584 (46.8) 579 (46.4) 85 (6.8)	275 (39.2) 416 (59.4) 10 (1.4)	134 (55.6) 99 (40.7) 9 (3.7)	194 (63.8) 108 (35.3) 3 (0.9)	<0.001*
**Duration, hrs** **3.0–3.9** **4**	20 (1.6) 1228 (98.4)	11 (1.6) 690 (98.4)	8 (3.3) 234 (96.7)	1 (0.3) 304 (99.7)	<0.001*
**Ultrafiltration volume, litres** **<2** **2.0–3.9** **≥4.0**	630 (50.5) 607 (48.7) 11 (0.8)	423 (60.3) 277 (39.5) 1 (0.2)	58 (24.1) 182 (75.0) 2 (0.9)	179 (58.6) 118 (38.8) 8 (2.6)	0.001*

IDH-intradialysis hypotension, IDHT-intradialysis hypertension, PreD-predialysis, BP-blood pressure, CKD-chronic kidney disease, CGN-chronic glomerulonephritis

* FET-Fisher's exact test, BFR-blood flow rate.
